# Never Change a Brewing Yeast? Why Not, There Are Plenty to Choose From

**DOI:** 10.3389/fgene.2020.582789

**Published:** 2020-11-06

**Authors:** Klaus B. Lengeler, Vratislav Stovicek, Ross T. Fennessy, Michael Katz, Jochen Förster

**Affiliations:** Carlsberg Research Laboratory, Carlsberg A/S, Copenhagen, Denmark

**Keywords:** Saccharomyces cerevisiae, breweries, diversity, reservoirs, strain collection, domestication

## Abstract

Fermented foods and particularly beer have accompanied the development of human civilization for thousands of years. *Saccharomyces cerevisiae*, the dominant yeast in the production of alcoholic beverages, probably co-evolved with human activity. Considering that alcoholic fermentations emerged worldwide, the number of strains used in beer production nowadays is surprisingly low. Thus, the genetic diversity is often limited. This is among others related to the switch from a household brewing style to a more artisan brewing regime during the sixteenth century and latterly the development of single yeast isolation techniques at the Carlsberg Research Laboratory in 1883, resulting in process optimizations in the brewing industry. However, due to fierce competition within the beer market and the increasing demand for novel beer styles, diversification is becoming increasingly important. Moreover, the emergence of craft brewing has influenced big breweries to rediscover yeast as a significant contributor to a beer’s aroma profile and realize that there is still room for innovation in the fermentation process. Here, we aim at giving a brief overview on how currently used *S. cerevisiae* brewing yeasts emerged and comment on the rationale behind replacing them with novel strains. We will present potential sources of yeasts that have not only been used in beer brewing before, including natural sources and sources linked to human activity but also an overlooked source, such as yeast culture collections. We will briefly comment on common yeast isolation techniques and finally touch on additional challenges for the brewing industry in replacing their current brewer’s yeasts.

## Introduction

While some anthropologists believe that the discovery to process foods over an open fire as much as 1.8 million years ago was crucial for the evolution of the human species, the early history of human societies is unquestionably linked to the domestication of plants and animals (Zeder, 2006), and the subsequent development of fermented foods during the Neolithic period (Tamang et al., 2020). Fermented beverages have been an important part of civilizations for economic and health reasons for thousands of years (Campbell-Platt, 1994; Vilela, 2019), and alcoholic beverages in particular played a vital role in their traditional culture and social life (Legras et al., 2007; Dietrich et al., 2012).

Beer is one of the oldest fermented beverages known in human history. Archeological remains and pictographic evidence of cereal based beer-like brews have been found across the Fertile Crescent, the oldest dating back to the twelfth millennium BC (Katz and Maytag, 1991; Michel et al., 1992; Hornsey, 2003; Black et al., 2006; Legras et al., 2007; Sicard and Legras, 2011; Liu et al., 2018). China also has a long history of beer-like beverages (McGovern et al., 2004; Wang et al., 2016), and in Europe, Celtic tribes spread beer brewing across the continent up to 2,000 years ago (Corran, 1975; Nelson, 2005), where over time it developed into the modern beer-brewing process as we know it today (Hornsey, 2003; Unger, 2004). While many changes have been made to the fermentation process over time, the one constant factor needed for successful beer production has been an alcohol producing yeast (Lodolo et al., 2008). However, despite the vast number of different beer styles that have been developed all over the world (Protz, 1995; Glover, 1997), the number of yeasts used in the brewing industry today is surprisingly low.

## Industrialization of Beer Production

For centuries beer production relied on spontaneous fermentations with complex mixtures of native/wild microbes, although yeasts belonging to the genus *Saccharomyces* usually dominate the later part of the process due to their strong ability to produce and tolerate ethanol (Steensels and Verstrepen, 2014). The early brewers had yet soon realized that repitching a new fermentation with a small portion of a just finished one resulted in faster and more reliable fermentations, a process commonly referred to as “backslopping” (Whittington et al., 2019). The constant reuse in man-made environments and the simultaneous reduction in contact to natural niches resulted in the domestication of brewer’s yeasts (Steensels et al., 2019) accompanied by a dramatic reduction in species involved in the brewing process. In Europe, this development was fueled during the middle ages by a switch from predominantly household fermentations to a more artisan brewing style, where taverns and monasteries became the predominant brewers. In addition, the introduction of new regulations in the sixteenth century standardized the brewing process and limited the number of ingredients used (the famous “Reinheitsgebot” from 1,516; Dornbusch, 1998; Hornsey, 2003), which improved beer quality but probably also reduced the numbers of yeasts used even further. Interestingly though, restricting the brewing period to the winter season not only resulted in a novel beer style commonly known as “lager” (vs. the traditional “ale,” Jentsch, 2007) but also in a novel brewing yeast lineage, that is, better adapted to lower fermentation temperatures (Unger, 2004; Dredge, 2019). During the industrial revolution of the nineteenth century, other groundbreaking technical discoveries were made that modernized the brewing process and allowed the year around production of “lager” beers. However, the most important discovery was made by the French scientist Louis Pasteur, who demonstrated that the splitting of malt sugar into alcohol and carbonic acid was due to the activity of a microbe, which is the brewer’s yeast (Pasteur, 1857, 1858). Inspired by these findings, Emil Christian Hansen developed techniques for isolation of single yeast cells at the Carlsberg Research Laboratory in the early 1880s, and the first pure yeast culture-based beer was brewed in 1883 (Hansen, 1888; Barnett and Lichtenthaler, 2001). From this time onward, the fast-growing beer industry began to replace spontaneous and “backslopping” based fermentations with its complex microbiomes by controlled fermentations using pure yeast starter cultures. Specifically, these were *S. cerevisiae* for “ale” and *S. pastorianus* for “lager” beers. Finally, while the number of microbreweries has increased since the late twentieth century, the global beer market is for the most part dominated today by only few internationally operating companies. In order to save costs and reduce complexity in the production processes, these big companies tend to reduce the already low number of brewing yeasts used within a company even further. Hence, human activity over the centuries resulted in a rather low number of specifically adapted yeasts used for beer brewing today (Gibson et al., 2007; Libkind et al., 2011; Steensels et al., 2014; Gallone et al., 2016).

## Toward More Yeast Diversity in Beer Brewing

Despite all industrialization and modernization efforts, the brewing industry is considered a rather traditional one, and breweries are usually quite reluctant to change their brewing yeasts. However, there has been an increasing demand for novel beer styles from customers and consumers over the last one or two decades, which has been mainly driven by the emergence of the craft brewing community. Since craft breweries usually operate in much smaller volumes, they are more willing to experiment with raw materials to create more “explorative” beer styles, basically reversing the streamlining of the brewing processes previously outlined. With increasing competition in beer markets, the entire brewing industry has been forced to reconsider their approach and diversify their beverage portfolios. In that context, not only craft breweries but also the big players “rediscovered” that yeast has just as much influence on beer quality but more importantly on beer taste as the other raw materials and/or physical brewing parameters (White and Zainasheff, 2010). Experimenting with novel yeasts, they realized that there is still a hidden treasure to be found not only from a product point of view, but also from a marketing perspective since doing business “with a story” is becoming more and more popular. Selling a beer brewed with a 5,000-year-old yeast (Aouizerat et al., 2019) is a marketing consultant’s dream. Therefore, it is an intriguing idea to go out and isolate novel yeasts and use them for brewing purposes.

## Isolation of Novel Brewing Yeast

**Figure 1:**
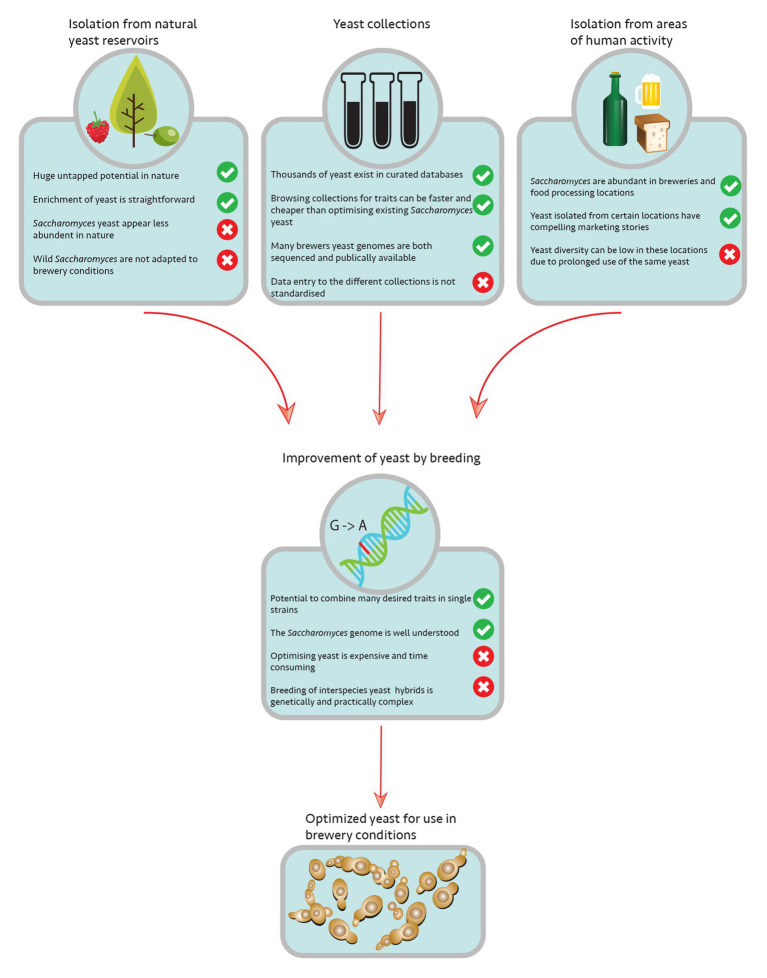
Overview of microbial reservoirs and strategies to introduce novel *Saccharomyces* diversity to breweries.

The most obvious sources for isolating yeast strains are the ones immediately linked to human *S. cerevisiae*-based activities (Figure 1), such as baking bread and fermenting wine or sake. Due to the high economic impact of these processes, a lot of research has been conducted on the yeasts involved in making these products (Ohya and Kashima, 2019; Parapouli et al., 2020). Several wine, sake, and baker’s yeasts have been analyzed for their beer brewing potential over the years. However, only few baker’s yeasts showed promising potential (Canonico et al., 2014; Marongiu et al., 2015; Rossi et al., 2018; Cubillos et al., 2019; Tokpohozin et al., 2019). The phylogenetic analyses of industrial *S. cerevisiae* strains in recent times have shown that most strains analyzed fell into a handful of clades and that were characterized by a certain production process (Gallone et al., 2016; Gonçalves et al., 2016; Kang et al., 2019). Further analyses could link these clades to genetic and phenotypic differences that most likely resulted from specifically adapting to the respective fermentation conditions, a phenomenon typical for domestication events (Driscoll et al., 2009; Purugganan and Fuller, 2009; Steensels et al., 2019). Interestingly, most baker’s yeast strains are closely related to their beer brewing counterparts, sharing several genetic and phenotypic features (Gonçalves et al., 2016), something that can be explained by the strong interconnection of baking and brewing in the early days. These analyses also confirmed a common “problem” for all these domesticated yeasts, a lack of genetic but sometimes also phenotypic variability especially within a clade. In fact, besides the fermenting microbe, these processes share quite a similar history. Except for sake fermentation, which was first mentioned in the eighth century, they emerged in the Neolithic period (Evans, 1990; McGovern et al., 1996, 1997; Cavalieri et al., 2003; Sicard and Legras, 2011). They were initially based on spontaneous fermentations, but have developed into highly engineered processes using only a small number of specialized pure yeast starter cultures since then (Heard and Fleet, 1985; Henick-Kling et al., 1998; Domizio et al., 2007; Daenen et al., 2008; Randez-Gil et al., 2013; Borneman et al., 2016). In order to avoid isolating yeast that is similar to what is already in use, one could potentially focus attention to more traditional fermentations, where indigenous yeasts may have escaped parts of the domestication processes and therefore provide a higher natural diversity. In addition, other natural substrates/raw materials than grains and grapes are sometimes used to create these traditional alcoholic beverages such as the honey-based mead, adding another level of complexity. Only in recent years have people started to look into the beer brewing potential of yeasts from traditional fermentations, such as cachaça spirits (Araújo et al., 2018), pulque, tequila (Cubillos et al., 2019), or sub-Saharan alcoholic beverages (Johansen et al., 2019). However, only the historically proven old-style beers such as Finnish sahti and the traditional Norwegian kveik beer, both using juniper instead of or in addition to hops, or the bread based Russian kvass have shown promising results so far (Dlusskaya et al., 2008; Ekberg et al., 2015; Preiss et al., 2018). There are still many traditional beverages left to explore such as traditional European low alcoholic beverages (Baschali et al., 2017) or fermented drinks produced by the Aboriginal people in Australia even before European arrival (Mangaitch, Way-a-linah, and Kambuda; Brady, 2008). Further research is needed to isolate and characterize yeast from these drinks. In addition to alcoholic beverages, other fermented foods could also serve as a target for novel yeast isolation. In 1987, Campbell-Platt reported the existence of around 3,500 different fermented foods and beverages, but there might be more than 5,000 varieties of these products being consumed in the world today (Campbell-Platt, 1987; Tamang, 2010). The role of *S. cerevisiae* in nonalcoholic fermentations has so far been less studied because among other things research is often complicated by the presence of other microorganisms, mainly lactic acid bacteria. Furthermore, *S. cerevisiae* could not only be identified in many dairy based fermentations but also in fermentations used to refine other agricultural products such as coffee or cocoa beans (Tofalo et al., 2019) that could be tested in beer fermentations. In addition, industrial bioethanol production has been reported to be a rich source of *S. cerevisiae* strains (Basso et al., 2008). Particularly, in Brazilian bioethanol plants, the specific operating conditions allow for the entry and growth of wild yeast strains (Amorim et al., 2011). The initial yeast starter cultures were replaced over time by indigenous strains present in sugar cane molasses that would outperform the domesticated yeasts due to their resistance to high ethanol levels and temperature fluctuations (Beato et al., 2016; Reis et al., 2017; Kechkar et al., 2019). Recently, several *S. cerevisiae* strains isolated from Brazilian distilleries have been investigated in high gravity beer fermentations (Christofoleti-Furlan et al., 2020), clearly showing they harbor a potential for use in the brewing industry.

## Yeast Origin and Diversity

As just exemplified by the Brazilian bioethanol production, the isolation of wild yeast strains from natural reservoirs could be potentially very interesting, as these could bear novel features useful for biotechnological applications (Figure 1). Even though *S. cerevisiae* is one of the most extensively studied organisms, it has only recently evolved into a model system for population genomic, biogeographic, and ecological studies (for review, see Alsammar and Delneri, 2020). Based on its metabolism, it was initially thought that *S. cerevisiae* would mainly reside on the surface of fruits and could therefore be found in respective environments, such as orchards and wineries. Surprisingly, in recent years, it was shown that the yeasts present in wineries seem mainly not to originate from grapes since their presence on intact grapes is rather scarce (Mortimer and Polsinelli, 1999; Taylor et al., 2014). Instead, it seems that yeast cells are most likely transferred to these ecosystems *via* insect vectors that feed off damaged grapes (Stefanini et al., 2012; Buser et al., 2014; Dapporto et al., 2016). The potential of insects as a natural reservoir for wild *S. cerevisiae* strains is still unclear and needs further investigation (Stevic, 1962). While some researchers believe that *S. cerevisiae* lives a nomadic life style since it can adapt to a wide variety of growth conditions (Goddard and Greig, 2015), more recent studies indicated that certain trees, especially from the order *Fagales* and the surrounding soil seem to be a natural niche for the organism (Hittinger, 2013). In fact, the most primitive *S. cerevisiae* populations to date, which showed a significantly higher genetic variability than previously isolated strains, were isolated from remote primeval forests in China (Wang et al., 2012; Boynton and Greig, 2014; Liti, 2015; Duan et al., 2018; Peter et al., 2018; Bendixsen et al., 2020). Even though *S. cerevisiae* is generally considered to be a terrestrial yeast, very few strains have been isolated from marine waters in coastal areas (Zaky et al., 2014) or freshwater lakes and rivers (Sláviková and Vadkertiová, 1995, 1997). Although the strains isolated from such habitats possess distinct phenotypical characteristics (Šťovíček et al., 2010, 2014), the question of whether these sites are an additional natural niche for *S. cerevisiae* or whether it only reflects human activity in the area, which needs further investigation. There are two main challenges when isolating wild *S. cerevisiae* strains. Due to the vast geographical distribution of strains linked to human activity, special care needs to be taken in choosing the right habitat during sampling to avoid isolating strains of the “common” genotypes. In addition, the low number of isolates in general poses another challenge to this approach asking for proper isolation techniques (Beech and Davenport, 1971). In a recent report, Boynton and colleagues discussed two of the most common isolation techniques, direct plating and so-called enrichment cultures, comparing their feasibility in isolating yeasts of the genus *Saccharomyces* from forest samples (Boynton et al., 2019). The results confirmed the general acceptance that enrichment culturing techniques favor the isolation of rare *Saccharomyces* species such as *S. cerevisiae*. However, since each enrichment protocol may select for certain strain features, sampling methods need to be carefully adjusted to interest and sampling habitats, the development of marine yeast isolation protocols being one example (Zaky et al., 2016). Besides the technical and biological challenges, the Convention of Biological Diversity (CBD) and its supplementary agreement, the Nagoya Protocol should be followed when isolating organisms from natural reservoirs. Proper documentation during the sampling process is required to ensure the conservation and sustainable use of biological diversity under this legal framework (Boundy-Mills et al., 2016).

## Yeast Culture Collections

While isolating and characterizing novel *S. cerevisiae* strains from the wild for industrial applications is an intriguing idea, only big breweries usually have the personnel and the technical requirements to run respective projects or are willing to outsource these tasks to scientific collaborators. Instead, most breweries will rely on a small number of commercially available yeasts that are provided by few companies specialized on brewing yeasts (Quain, 2006). However, another invaluable source of yeasts strains seems to have been overlooked in the brewing industry for a long time, such as yeast culture collections (Figure 1; Daniel and Prasad, 2010). This comes as a little surprise since one of first culture collections, though not publicly available, was established at the Carlsberg brewery shortly after Hansen had developed the pure culture isolation techniques (Hansen, 1888; Barnett, 2007). Ever since the first service culture collections were established around 1,900 (Uruburu, 2003), the number of culture collections has increased significantly, ranging from well-known, large public repositories to dozens of smaller public collections. Even though the collections are generally considered to represent only a small fraction of the microbial diversity (Boundy-Mills, 2012), they can be an invaluable source of well-characterized strains that were isolated from various natural or human activity associated niches. In addition, especially the larger collections have started to offer scientific services, which could be another option for breweries to outsource prospective projects. Boundy-Mills and colleagues recently published a detailed analysis of modern yeast culture collections listing the most important collections and illustrating the challenges and opportunities for their users (Boundy-Mills et al., 2016).

## *Saccharomyces* Hybrids and Beyond

In this mini review, we have focused on *S. cerevisae*. However, it should be mentioned that interspecies hybrids between *S. cerevisiae* and usually another member of the *Saccharomyces* genus play a significant role in industrial fermentation processes including beer brewing. For example, it was found that up to 25% of the top fermenting yeasts isolated from Belgian Lambic/Trappist beer production are interspecies hybrids between *S. cerevisiae* and *S. kudriavzevii* (González et al., 2008; Peris et al., 2012, 2018). More importantly, the previously mentioned *S. pastorianus* “lager” yeast, responsible for 90% of the beer produced worldwide today, was suggested to be a hybrid already decades ago (for an early review, see Polaina, 2002). However, it was only in 2011 that the missing non-*S. cerevisiae* parent of the “lager” yeast lineage was isolated and identified as *S. eubayanus* (Libkind et al., 2011). Hybrids often show a better performance under certain conditions since they combine features otherwise unique to one of the parental species, e.g., the high fermentative capacity of *S. cerevisiae* and the cold tolerance of *S. kudriavzevii* or *S. eubayanus*, resulting in hybrids suitable for cold temperature fermentations (Mertens et al., 2015; Krogerus et al., 2017; Nikulin et al., 2018; Ortiz-Tovar et al., 2018; Baker et al., 2019). In addition, hybrids can also benefit from a less well-understood effect called heterosis or hybrid vigor, where the resulting hybrid shows a greater fitness than one would expect based on each parental strain (Lindegren et al., 1953; Hebly et al., 2015; Bernardes et al., 2017). Although hybrids have a huge potential for industrial use, they themselves are usually less suitable for performing genetic crosses since most strains will suffer from hybrid sterility, resulting in very low numbers of viable progeny with a clear mating type (Sipiczki, 2018, 2019; Ono and Greig, 2020; Sipiczki et al., 2020). While there were attempts to create novel “lager” strains by crossing meiotic offspring of “lager” yeasts already in the early 1980s (Gjermansen and Sigsgaard, 1981), it was the identification of *S. eubayanus* that changed the concept of creating novel “lager” yeasts significantly. Once *S. eubayanus* was available novel *S. cerevisiae* × *S. eubayanus* hybrids could be generated by “simple” interspecies crosses, and several reports over the last few years have clearly indicated the high potential of these novel hybrids (Krogerus et al., 2015; Mertens et al., 2015; Nikulin et al., 2018; Langdon et al., 2019). In addition, not only the increasing number of strains but also species within the *Saccharomyces* genus might facilitate the generation of a wider selection of intra- and inter-species hybrids with hopefully exciting novel features (Nikulin et al., 2018). Finally, as mentioned in the beginning, the beer brewing process was initiated by spontaneous fermentation for centuries, and still is for certain beer types today. A vast number of so-called nonconventional yeasts have been isolated from these mixed fermentations over the years and their impact on fermentation performance and flavor profiles has been studied (Spencer et al., 2002; Michel et al., 2016a; Varela, 2016; Gibson et al., 2017; Capece et al., 2018b; Holt et al., 2018; Canonico et al., 2019; Colomer et al., 2019; Cubillos et al., 2019). In addition to using pure yeast cultures from these nonconventional yeast species in brewing of novel beer styles or special beers (Steensels et al., 2015; Michel et al., 2016b; Capece et al., 2018a; De Francesco et al., 2018; Gutiérrez et al., 2018; Ravasio et al., 2018; Colomer et al., 2020; Iattici et al., 2020; Zdaniewicz et al., 2020), these yeasts could be used, where genetically possible, to expand the concept of novel interspecies hybrids for the brewing industry beyond the *Saccharomyces* genus adding even more genetic diversity to brewer’s yeasts.

## Outlook

Since prehistoric times, humans have used *S. cerevisiae* to convert sugar containing raw materials into flavorful alcoholic beverages, such as beer (Michel et al., 1992; Piskur et al., 2006; Steensels and Verstrepen, 2014). At the same time the organism has been domesticated over the span of centuries, resulting in the evolution of highly specific strain lineages optimized for beer brewing but dramatically reduced in genetic variability (McMurrough et al., 1996; Brown et al., 2010; Steensels and Verstrepen, 2014; Gallone et al., 2016; Gonçalves et al., 2016). While it is an attractive idea to breathe new life into current brewer’s yeasts by introducing novel wild yeasts into industrial applications, their use may not be as straight forward as anticipated. Most natural isolates will lack features that are necessary for the industrial scale beer production and the genetic diversity may not always correspond to a phenotypic one (Camarasa et al., 2011). This implies that strains will need to be improved (Figure 1). However, due to consumer concerns, the use of modern genetic approaches is usually objected (Bamforth, 2006; Saerens et al., 2010). Therefore, applied researchers currently rely on classic yeast breeding techniques. Interestingly enough that these techniques were initially developed by the brewing industry, when Øjvind Winge discovered the sexual cycle of *S. cerevisiae* and used his knowledge to combine desirable brewing traits by crossing different strains at the Carlsberg Research Laboratory in the 1930s (Winge, 1935; Barnett, 2007). So the brewing industry could end up renewing their applied yeast research and their strain portfolio by going back to their own roots (Gibson et al., 2017; Iattici et al., 2020).

## Author Contributions

KL and VS were the main contributors to the abstract, main text, and reference list. RF provided the Figure. MK and JF were involved in the initial planning of the manuscript and provided helpful comments throughout the preparation of the manuscript. All authors contributed to the article and approved the submitted version.

### Conflict of Interest

All authors of this article are employed by the company Carlsberg A/S, Copenhagen, Denmark. VS is funded through the “Beer Fingerprinting Project” grant no. 7045-00014A of the Innovation Fund Denmark.
